# Oxidative Stress and Inflammation Are Associated with Coexistent Severe Multivessel Coronary Artery Stenosis and Right Carotid Artery Severe Stenosis in Elderly Patients

**DOI:** 10.1155/2021/2976447

**Published:** 2021-12-22

**Authors:** Xia Li, Dianxuan Guo, Youdong Hu, Ying Chen

**Affiliations:** Department of Geriatrics, The Affiliated Huaian Hospital of Xuzhou Medical University, Huaian 223002, China

## Abstract

Oxidative stress and inflammatory response are the main pathogenic pathways in atherosclerosis stenosis. This study is aimed at evaluating the roles of oxidative stress and inflammatory response in coexistent right carotid artery severe stenosis and severe multivessel coronary artery stenosis in elderly patients. Circulating levels of total oxidant status (TOS), lipid hydroperoxide (LHP), 8-isoprostane (8-IP), malondialdehyde (MDA), monocyte chemotactic protein-4 (MCP-4), amyloid A (AA), high-sensitivity C-reactive protein (hs-CRP), and tumor necrosis factor-*α* (TNF-*α*) were measured by standardised laboratory test methods. Markers of oxidative stress and inflammatory response: levels of TOS, LHP, 8-IP, MDA, MCP-4, AA, hs-CRP, and TNF-*α*, were increased (*P* < 0.001) in elderly patient. These results suggested that oxidative stress and inflammatory response may be involved in carotid artery severe stenosis and severe multivessel coronary artery stenosis and measuring oxidative stress and inflammation biomarkers may also be a promising step in the development of an effective method for monitoring the severity of right carotid artery stenosis and multivessel coronary artery stenosis in elderly patients.

## 1. Introduction

Oxidative stress is related to intracranial carotid artery atherosclerosis, and the elevated cerebrovascular oxidative stress reduces cerebrovascular superoxide dismutase activity and promotes cerebrovascular oxidative injuries and intracranial carotid artery atherosclerosis [1, 2]. The vascular inflammatory response is associated with the development and progression of atherosclerotic plaques in the human aorta, carotid, cerebral, and coronary arteries [3]. The intracranial artery atherosclerosis is the most common cause of cerebral ischemic stroke [4].

Total oxidant status (TOS) is a marker of oxidative stress. High level of TOS inhibits expression of antioxidant enzymes and is associated with oxidant/antioxidant imbalance [5] and oxidant-antioxidant imbalance is involved in the development of atherosclerosis [6]. Lipid hydroperoxide (LHP) is a biomarker of oxidative stress. Histopathological studies show significant elevation in LHP induces the progression of oxidative damage [7] and accelerates the development and progression of atherosclerosis [8]. Increased 8-isoprostane (8-IP) as an oxidative damage biomarker induces oxidative stress and plays a key role in the progression of atherosclerosis [9, 10] and is further related to CAD and the extent of coronary stenosis [9]. High level of malondialdehyde (MDA) as reliable biomarker of oxidative stress induces oxidative stress and contributes to the imbalance between oxidant and antioxidant status and promotes the progression of atherosclerosis [11, 12]. Monocyte chemotactic protein-4 (MCP-4) as a proinflammatory molecular plays a key in atherogenesis. MCP-4 is expressed in the artery atherosclerotic lesions in patients with atherosclerotic coronary arteries and is involved in the atherosclerotic inflammatory process [13]. Amyloid A (AA) as an indicator of inflammatory response plays a key role in acute and chronic inflammatory response [14], and atherosclerosis is a chronic inflammatory response related to increased expression of AA in humans [15]. High-sensitivity C-reactive protein (hs-CRP) is a marker of systemic inflammatory response and inflammatory response plays an important role in atherosclerosis initiation and progression [16]. The pathogenic activity of proinflammatory cytokine tumor necrosis factor-*α* (TNF-*α*) promotes inflammatory response and plays a pivotal role in the progression of in atherosclerostic cardiovascular diseases [17]. This study is aimed at evaluating the relationships among oxidative stress and inflammation markers as well as carotid and coronary artery severe stenosis to test the contribution of oxidative stress and inflammation to coexistent right carotid artery severe stenosis and severe multivessel coronary artery stenosis in patients.

## 2. Material and Methods

### 2.1. Patient Selection

From 3 January 2014 to 25 December 2018, our research included the patients with carotid artery stenosis and carotid artery stenosis+coronary artery stenosis in different aged groups. The inclusion criteria adopted in the present research were (1) the patients aged 65 to 86 years old and (2) the patients with right common carotid artery stenosis (RCCAS), right external carotid artery stenosis (RECAS), right internal carotid artery stenosis (RICAS), and RCCAS+RECAS+RICAS+coronary artery stenosis. The research was approved by the Xuzhou Medical University and the University Human Research Ethics Committee according to the relevant laws of China, and the written informed consents were obtained for all participants according to the Revised Declaration of Helsinki. The patients with one or more of the following criteria were excluded: (1) acute and chronic brain infarction, (2) acute myocardial infarction, (3) upper limb artery stenosis or occlusion, (4) lower extremity arterial stenosis or occlusion, (5) coronary artery occlusion, (6) malignant tumors, (7) using antiphlogistic drugs, and (8) using antioxidants.

### 2.2. Research Protocol

The healthy individuals defined as without any illnesses [18] were included in the control (CON) group (*n* = 61) and patients without carotid stenosis defined as arterial intimal hyperplasia [19] were included in the without carotid stenosis group (*n* = 56). The numbers of patients with RCCAS, RECAS, RICAS, RCCAS+RECAS+RICAS, and RCCAS+RECAS+RICAS+coronary artery stenosis were 167, 162, 167, 165, and 155, respectively. The patients with RCCAS were included in mild right common carotid artery stenosis (MI-RCCAS) defined as the stenosis diameter < 50% [20] group (*n* = 59), moderate right common carotid artery stenosis (MO-RCCAS) defined as the stenosis 50-69% [20] group (*n* = 55), and severe right common carotid artery stenosis (SE-RCCAS) defined as the stenosis 70-99% [20] group (*n* = 53). The patients with RECAS were included in the mild right external carotid artery stenosis (MI-RECAS) group (*n* = 54), moderate right external carotid artery stenosis (MO-RECAS) group (*n* = 51), and severe right external carotid artery stenosis (SE-RECAS) group (*n* = 57). The patients with RICAS were included in the mild right internal carotid artery stenosis (MI-RICAS) group (*n* = 57), moderate right internal carotid artery stenosis (MO-RICAS) group (*n* = 54), and severe right internal carotid artery stenosis (SE-RICAS) group (*n* = 56). The numbers of patients with SE-RCCAS+SE-RECAS, SE-RCCAS+SE-RICAS, and SE-RCCAS+SE-RECAS+SE-RICAS were 58, 55 and 52, respectively. The numbers of patients with SE-RCCAS+severe one-vessel coronary stenosis (SOVCS), SE-RECAS+severe two-vessel coronary stenosis (STVCS), and SE-RICAS+severe multivessel coronary stenosis (SMVCS) were 51, 50, and 54, respectively. Severe coronary stenosis was defined as ≥70% narrowing [21].

### 2.3. Evaluations of Carotid Stenosis and Coronary Artery Stenosis

The assessment of carotid stenosis was measured with computed tomography angiography and Color Doppler ultrasonography [22]. Coronary artery stenosis was determined on both stress and rest coronary computed tomography angiography images [23] The evidence was assessed independently by three experienced interventional cardiologists.

### 2.4. Measurements of the Levels of TOS, LHP, 8-IP, and MDA

Fasting venous blood samples were collected into blood test tubes, and the serum was separated from blood samples by centrifugating at 1500 g for 15 min at 4°C, and the blood serum samples were stored frozen at -80°C until further TOS analysis [24]. The evaluations of LHP in the patients' plasma were performed by using ferrous oxidation in xylenol orange assays in conjunction with triphenylphosphine, and the results were expressed as *μ*mol/L [25]. Blood plasma samples were collected and centrifuged at 6000xg for 15 minutes at 4°C and stored frozen immediately at -80°C until further 8-IP analysis. Concentrations of 8-IP analyses were determined by using enzyme immunoassay (ELISA), and all results were expressed as pg/mL [26]. The concentration of MDA was measured by using ELISA, which was performed by using a commercial ELISA kit for the measurements of the levels of MDA (DL Naturegene Life Sciences, Inc., USA). All serum samples were stored frozen at -80°C until analysis. Reading of the results was measured at 456 nm on a plate reader (STAT FAX 2100, Awareness technology Inc., USA). The concentrations of MAD were expressed in nmol/L [27].

### 2.5. Measurements of the Levels of MCP-4, AA, hs-CRP, and TNF-*α*

After overnight fasting, the venous blood samples were collected and frozen immediately at -80°C until further use. The levels of serum MCP-4 were measured by ELISA assays (R&D Systems, Minneapolis, MN, USA) according to the manufacturer's instruction [28]. The serum samples were collected and frozen immediately and kept at -80°C until further processing. AA in serum was quantified by using ELISA kit from Human AA Assay Kit (Invitrogen Life Technologies, Carlsbad, CA, USA) according to the instruction of the kit [29]. Blood plasma samples were collected from participants after overnight fasting and were stored frozen at -80°C until use. The hs-CRP in plasma was determined by turbidometric assay and ELISA method (ELISA kit, Calbiotech Inc., Spring Valley, CA, USA) [30]. Venous serum blood samples were collected after overnight fasting and were stored frozen in aliquots at -80°C until further processing. Sensitive ELISA sandwich immunoassay (Immundiagnostik AG, Bensheim, Germany) was used for determining TNF-*α* according to manufacturer's instructions [31].

### 2.6. Data Analysis

Normality of the variables was assessed using the Shapiro-Wilks test to determine whether the variables fit the normal distribution. All *P* values for Shapiro-Wilks tests were greater than 0.05, which indicated the normality of all variables. The data were presented as mean ± standard deviation for normally distributed values. Because serum and plasma levels of TOS, LHP, 8-IP, MDA, MCP-4, AA, hs-CRP, and TNF-*α* were assay data, paired Student's *t*-tests were used to calculate the statistical comparisons between each pair of groups and the one-way analysis of variance (ANOVA) was applied to examine the variances of the different groups. The multiple regression method was employed to determine the independent risk factors for the carotid artery severe stenosis and severe coronary artery stenosis. The statistical versus clinical significances in different study groups were achieved when *P* values were <0.05. The tolerance and variance inflation factor (VIF) were used to assess collinearity between variables, with tolerance < 0.1 and VIF > 10 considered indicative of collinearity. The tolerance was >1, and VIF was significantly <10 for the variables, indicating no collinearity among the all independent variables. The statistical analyses of the participant's clinical data were analysed using the SPSS (24.0) statistical analysis package software programs for conducting statistical analysis (SPSS, An IBM Corporation, Armonk, NY, USA) in the evaluations of different levels of TOS, LHP, 8-IP, MDA, MCP-4, AA, hs-CRP, and TNF-*α*.

## 3. Results

### 3.1. The Participant Baseline Characteristics

The characteristics of individuals participating were very similar among different study groups (Table 1). All participants in study groups were well-matched without significant differences in gender, age, coronary artery disease (CAD) defined as coronary arteries with stenosis ≥ 50% in a major epicardial coronary arteries [32], hypertension defined as blood pressure higher than 130/80 mmHg [33], diabetes mellitus defined as fasting plasma glucose ≥ 126 mg/dL (7 mmol/L) [34], history of stroke defined as intracranial ischemia and hemorrhage [35], current smoking defined as >20 pack-years and >20 joint-years, respectively [36], current drinking defined as any alcoholic drinking and alcoholic binge drinking as consuming five or more drinks on one or more occasion, both in the past two months [37], regular exercise defined as any kind of favorite recreational or sport and physical activity other than simply walking more than three days a week for at least twenty minutes [38], myocardial infarction defined as sudden ischemic necrosis of myocardial tissue [39], dizziness defined as persistent subjective unsteadiness [40], memory impairment defined as delayed word recall test score < 4 and poor cognitive function by mini-mental state examination score < 25 [41], amaurosis fugax defined as transient monocular vision loss secondary to the retinal ischemia [42], cognitive impairment defined as impairment of ≥2 cognitive tests [43], and angina pectoris defined as pain, pressure, or discomfort in the chest [44].

### 3.2. Levels of TOS, LHP, 8-IP, MDA, MCP-4, AA, hs-CRP, and TNF-*α* in Elderly Patients with MI-RCCAS, MO-RCCAS, and SE-RCCAS

The levels of TOS, LHP, 8-IP, MDA, MCP-4, AA, hs-CRP, and TNF-*α* were increased significantly in the SE-RCCAS group when compared with the MI-RCCAS and MO-RCCAS groups, respectively (*P* < 0.001). It is likely that the increased levels of oxidative stress and proinflammatory response may play a key role in the development of right common carotid artery stenosis (Table 2).

### 3.3. Oxidative Stress and Proinflammatory Response Were Associated with MI-RECAS, MO-RECAS, and SE-RECAS in Elderly Patients

The levels of TOS, LHP, 8-IP, MDA, MCP-4, AA, hs-CRP, and TNF-*α* were increased significantly in the SE-RECAS group compared to the MI-RECAS and MO-RECAS groups, respectively (*P* < 0.001). Thus, inhibition of oxidative stress and proinflammatory response in elderly patients with right external carotid artery stenosis may be beneficial (Table 3).

### 3.4. Biomarker Levels of Proinflammation and Oxidative Stress in Elderly Patients with MI-RICAS, MO-RICAS, and SE-RICAS

Levels of TOS, LHP, 8-IP, MDA, MCP-4, AA, hs-CRP, and TNF-*α* were increased significantly in the SE-RICAS group compared to the MI-RICAS and MO-RICAS groups, respectively (*P* < 0.001) (Table 4). Our study demonstrated that high levels of pro-inflammation and oxidative stress may be related to RICAS.

### 3.5. Levels of Proinflammation and Oxidative Stress in Elderly Patients with SE-RCCAS+SE-RECAS, SE-RCCAS+SE-RICAS, and SE-RCCAS+SE-RECAS+SE-RICAS

The levels of TOS, LHP, 8-IP, MDA, MCP-4, AA, hs-CRP, and TNF-*α* were increased significantly in the SE-RCCAS+SE-RECAS+SE-RICAS group compared to the SE-RCCAS+SE-RECAS and SE-RCCAS+SE-RICAS groups, respectively (*P* < 0.001). Our study demonstrated that the interplay of oxidative stress and inflammation may promote the development of carotid artery severe stenosis in patients (Table 5).

### 3.6. The Levels of Proinflammation and Oxidative Stress in Elderly Patients with SE-RCCAS+SOVCS, SE-RECAS+STVCS, and SE-RICAS+SMVCS

The levels of TOS, LHP, 8-IP, MDA, MCP-4, AA, hs-CRP, and TNF-*α* were increased significantly in the SE-RICAS+SMVCS group compared to the SE-RCCAS+SOVCS and SE-RECAS+STVCS groups, respectively (*P* < 0.001) (Table 6). We also observed that the expression of markers of inflammation and oxidative stress was responsible for carotid artery severe stenosis and severe coronary artery stenosis. Increased oxidative stress and inflammation may cause the initiation and progression of carotid artery severe stenosis and severe coronary artery stenosis (Table 6).

### 3.7. Incidences of Carotid Artery Severe Stenosis and Severe Coronary Artery Stenosis in Elderly Patients

The incidences of SE-RAS+SMVCS in 81-86 years old age group were higher than 65-70 and 75-80 years old age groups. These results showed that SE-RAS+SMVCS occurred more frequently in 81-86 years old age group than in 65-70 and 75-80 years old age groups (Table 7).

### 3.8. Multiple Regression Analysis to Calculate and Confirm the Statistical Significance of Variables for Carotid and Coronary Artery Stenosis

By multiple regression analyses, TOS, LHP, 8-IP, MDA, MCP-4, AA, hs-CRP, and TNF-*α* were found to be independent risk predictors of worsening of carotid and coronary artery lesions after adjustment for gender, age, CAD, hypertension, diabetes mellitus, history of stroke, current smoking, current drinking, regular exercise, myocardial infarction, dizziness, delayed memory, amaurosis fugax, and angina pectoris in elderly patients. All *P* values of less than 0.05 were regarded as statistically significant (Table 8).

## 4. Discussion

The increased TOS as oxidative stress marker leads to oxidative stress, resulting from an imbalance of reactive oxygen species-antioxidants [45]. Oxidative stress causes oxidative damage in vascular endothelium, leading to vascular endothelial cell dysfunction and cardiovascular diseases and plays a key role in the progression and development of atherosclerosis [46]. High levels of LHP are related to an imbalance between generation and removal of free radicals, promoting oxidative vascular damages, vascular dysfunction, oxidative damage to cell proteins, cell membrane, and cellular nucleic acids [47]. 8-IP, a prostaglandin-like substance produced through cyclooxygenase-independent mechanisms, is considered one of the most sensitive and reliable biomarker of oxidative stress as indicator of oxidative stress status [48]. MDA as a biomarker of oxidative stress is involved in the initiation and the development of atherosclerosis [49]. MDA is formed during the lipid peroxidation, leading to generation of oxidative stress and interplay with cell membrane receptors promoting the inflammatory procession and arterial plaque ruptures [50]. We showed levels of oxidative stress (TOS, LHP, 8-IP, and MDA) were increased in elderly patients with coexistent right carotid artery severe stenosis and severe multivessel coronary artery stenosis. Our study indicated that the increased levels of oxidative stress showed significant correlation with carotid artery severe stenosis and severe multivessel coronary artery stenosis in elderly patients and demonstrated that the oxidative stress may promote the development and progression of carotid artery severe stenosis and severe multivessel coronary artery stenosis in elderly patients. This indicated that high oxidative stress level played key role in worse prognosis of carotid artery stenosis and multivessel coronary artery stenosis in these patients.

MCP-4 is related to low-density lipoprotein-cholesterol and an independent prognostic predictor of elevated carotid arterial intima-media thickness. MCP-4 plays a role in atherosclerosis by increasing the circulating levels of the macrophage inflammatory protein-1 beta and is the main atherosclerosis and inflammatory biomarkers [51, 52]. AA mRNA is detected in atherosclerotic plaques in human arteries, and AA gene is related to carotid artery intimal media thickness. Inflammatory response is central to AA pathophysiology and development of atherosclerosis [15], and inflammatory response is the important component of atherosclerotic plaques. AA as an inflammatory marker is associated with inflammatory response and atherosclerosis, and the persistently increased AA level is involved in acute and chronic inflammatory injuries. The hs-CRP promotes inflammatory response in the progression of atherosclerosis and plays an important role in atherosclerosis [53]. TNF-*α* significantly increases the vascular endothelial inflammatory response and atherosclerosis through nuclear factor kappa-B signaling. Atherosclerosis is an inflammatory process of the artery walls and TNF-*α* as a proinflammatory cytokine triggers nuclear factor kappa-B inflammatory pathway and is involved in an accelerated development of vascular endothelial inflammation and atherosclerosis [54]. We identified relevant inflammatory biomarkers (MCP-4, AA, hs-CRP, and TNF-*α*) of severe carotid artery stenosis and coronary artery stenosis and toxicological effects of inflammatory response on carotid artery stenosis and multivessel coronary artery stenosis in elderly patients. Oxidative stress is a key factor that triggers inflammatory response [55], and therefore, the high levels of oxidative stress and inflammatory response may simultaneously resulted in the progression of carotid artery stenosis and coronary artery stenosis in elderly patients.

Increased oxidative stress promotes inflammatory response, and the complex interplay between oxidative stress and inflammatory response plays a key role in the pathogenesis of disease [55]. The elevation of intracellular oxidative stress levels and the upregulation of the expression of proinflammatory genes lead to a series of cellular and molecular events (serious cellular toxicity and apoptosis) [56]. Inflammatory response directly causes cellular toxicity, apoptosis, and necrosis through oxidative stress, and interplay of oxidative stress and inflammatory response leads to the activation of oxidative stress and the increased levels of pro-inflammatory mediators such as TNF-*α* [56] and interleukin-15 as an inflammatory cytokine independently associated with CAD and carotid intima-media thickness, suggesting a main role of IL-15 in the atherosclerosis process [57]. Clinical trials have revealed crosstalk between oxidative stress and inflammatory response is closely associated with atherosclerosis, and this close link is also supported by reports on aggravated proinflammatory phenotype or overgeneration of reactive oxygen species [58]. In this study, we assessed the relationships between oxidative stress, inflammatory response, and right carotid artery severe stenosis as well as severe multivessel coronary artery stenosis in elderly patients. We found that elderly patients with carotid and coronary artery severe stenosis had higher levels of oxidative stress and inflammatory response in our studies. We also found that oxidative stress and inflammatory response were associated with right carotid artery severe stenosis and severe multivessel coronary artery stenosis in elderly patients. Moreover, we found that 81 to 86 years old patients suffered from coexistent severe multivessel coronary artery stenosis and right carotid artery severe stenosis more easily. The association among oxidative stress, inflammatory response, and carotid as well as coronary artery severe stenosis provided evidences for the potential mechanisms of carotid and coronary artery severe stenosis in elderly patients. The mechanisms could be considered that continuous high levels of oxidative stress were involved in the initiation and progression of carotid and coronary artery severe stenosis; the expressions of inflammatory mediators played an important role in carotid and coronary artery severe stenosis; the oxidative stress regulated inflammatory processes and initiated a process of inflammatory response [57], indicating that the interplay between oxidative stress and inflammatory response further accelerated development and progression of carotid and coronary artery severe stenosis in elderly patients.

Right carotid artery severe stenosis leads to cerebral circulatory insufficiency, intracerebral steal phenomenon [59], reduction of blood flow of the right cerebral hemisphere, hemichorea [60], transient ischemic attacks, and strokes [61], and the rates of concurrent carotid artery stenosis and CAD are elevating gradually day by day. CAD patients who are undergoing percutaneous coronary intervention have severe carotid artery stenosis and the majority of patients with carotid artery severe stenosis have complex CAD [62]. The severe multivessel coronary artery stenosis as a risk factor is related to severe carotid artery stenosis [62]. Patients with concurrent carotid and coronary artery severe stenosis are demonstrated to have a more severe atherosclerosis involving multiple arteries [62]. Therefore, it is very important clinically for studying concurrent right carotid artery severe stenosis and severe multivessel coronary artery stenosis in elderly patients.

## 5. Limitations

The findings from our research had several limitations. The lifestyle in elderly patients with coronary artery and carotid artery severe stenosis was not evaluated in the present study and life style factors including body mass index, nutrition condition, dietary habits, and exercise may influence the coronary and carotid atherosclerosis through oxidative stress and inflammation. Our data were also limited in lacking assessment of the effects of drinking patterns (none to moderate drinking, nonproblematic heavy drinking, and problem drinking) on blood biomarkers of oxidative stress and inflammatory mediators in elderly patients with severe coronary and carotid stenosis. Another limitation was lack of research data on the changes of oxidative stress and inflammation markers in active and passive smoking that could have better clarified the relationships among oxidative stress, inflammation, the progression of coronary atherosclerosis, and severe carotid atherosclerosis. Besides, there was no information available regarding the impacts of comorbidities (e.g., type 2 diabetes mellitus, hypertension, and angina pectoris) on oxidative stress and inflammation in elderly patients with severe coronary and carotid stenosis. It would have more information if we could examine the detailed histopathological characterizations of atherosclerotic plaques in the coronary and carotid arteries.

## 6. Conclusions

This research shows that high levels of TOS, LHP, 8-IP, MDA, MCP-4, AA, hs-CRP, and TNF-*α* are associated with severe coronary and carotid stenosis suggesting a possible roles of oxidative stress and inflammatory mediators in the development of coronary and carotid atherosclerosis. Further research with larger sample sizes and adequate follow up period will be required to better understand the mechanisms of severe coronary and carotid atherosclerosis in elderly patients.

## Figures and Tables

**Table 1:** Baseline characteristics of patients with RCCAS, RECAS, RICAS, RCCAS+RECAS+RICAS, and RCCAS+RECAS+RICAS+coronary artery lesions.

	CON (*n* = 61)	No carotid stenosis (*n* = 56)	RCCAS (*n* = 167)	RECAS (*n* = 162)	RICAS (*n* = 167)	RCCAS+RECAS+RICAS (*n* = 165)	RCCAS+RECAS+RICAS+coronary artery stenosis (*n* = 155)	*P* value
Gender								
Male, *n* (%)	31 (51)	29 (52)	83 (49)	82 (51)	83 (49)	82 (49)	79 (51)	0.41
Female, *n* (%)	30 (49)	27 (48)	84 (51)	80 (49)	84 (51)	83 (51)	76 (49)	0.43
Age (years)	65.1 ± 13.0	65.8 ± 13.2	64.2 ± 13.4	65.1 ± 14.0	63.8 ± 14.4	62.2 ± 15.1	76.6 ± 15.3	0.32
CAD, *n* (%)	0	17 (30)	83 (49)	81 (50)	84 (50)	82 (49)	77 (50)	0.41
Hypertension, *n* (%)	0	15 (27)	35 (21)	54 (33)	60 (35)	54 (32)	47 (30)	0.07
Diabetes mellitus, *n* (%)	0	10 (18)	33 (19)	30 (18)	33 (20)	32 (19)	30 (19)	0.12
History of stroke, *n* (%)	0	0	9 (5)	8 (5)	7 (4)	8 (4)	9 (5)	0.06
Current smoking, *n* (%)	0	6 (11)	42 (25)	29 (17)	40 (23)	29 (17)	40 (25)	0.07
Current drinking, *n* (%)	0	0	9 (5)	11 (7)	9 (4)	12 (7)	9 (5)	0.06
Regular exercise, *n* (%)	5 (8)	4 (7)	7 (4)	5 (3)	6 (3)	4 (2)	9 (6)	0.17
Myocardial infarction, *n* (%)	0	0	0	0	0	0	11 (7)	0.14
Dizziness, *n* (%)	0	0	6 (3)	3 (1)	4 (2)	7 (4)	4 (2)	0.10
Memory impairment, *n* (%)	0	0	25 (15)	21 (13)	17 (10)	20 (12)	9 (6)	0.27
Amaurosis fugax, *n* (%)	0	0	16 (9)	13 (8)	12 (7)	11 (6)	10 (6)	0.16
Cognitive impairment, *n* (%)	0	0	10 (5)	7 (4)	6 (3)	8 (4)	7 (4)	0.09
Angina pectoris, *n* (%)	0	0	0	0	0	0	20 (13)	0.14

CON: control; RCCAS: right common carotid artery stenosis; RECAS: right external carotid artery stenosis; RICAS: right internal carotid artery stenosis; CAD: coronary artery disease.

**Table 2:** Levels of biomarkers in patients with RCCAS.

	CON (*n* = 61)	No carotid stenosis (*n* = 56)	MI-RCCAS (*n* = 59)	MO-RCCAS (*n* = 55)	SE-RCCAS (*n* = 53)
TOS (*μ*mol H_2_O_2_ Eq/L)	7.3 ± 0.7	7.4 ± 0.7	7.8 ± 1.5^∗^	8.3 ± 0.8^∗∗^	9.1 ± 0.9^∗∗∗^
LHP (*μ*mol/L)	5.8 ± 0.6	5.9 ± 0.6	6.7 ± 0.7^∗^	7.9 ± 0.8^∗∗^	8.7 ± 0.9^∗∗∗^
8-IP (pg/mL)	45.2 ± 4.5	45.0 ± 4.4	56.2 ± 5.6^∗^	68.8 ± 6.9^∗∗^	77.4 ± 7.6^∗∗∗^
MDA (nmol/L)	1.7 ± 0.3	1.6 ± 0.2	2.8 ± 0.3^∗^	3.6 ± 0.4^∗∗^	4.5 ± 0.5^∗∗∗^
MCP-4 (ng/mL)	41.9 ± 4.2	42.0 ± 4.1	49.7 ± 5.0^∗^	58.5 ± 5.9^∗∗^	66.3 ± 6.6^∗∗∗^
AA (mg/L)	35.2 ± 3.5	36.1 ± 3.6	44.2 ± 4.3^∗^	57.0 ± 5.6^∗∗^	65.2 ± 6.5^∗∗∗^
hs-CRP (mg/L)	2.7 ± 0.3	2.6 ± 0.4	3.9 ± 0.4^∗^	5.6 ± 0.6^∗∗^	6.5 ± 0.7^∗∗∗^
TNF-*α* (ng/L)	20.1 ± 2.0	21.3 ± 2.1	29.4 ± 2.9^∗^	45.7 ± 4.6^∗∗^	50.6 ± 5.1^∗∗∗^

RCCAS: right common carotid artery stenosis; CON: control; MI-RCCAS: mild right common carotid artery stenosis; MO-RCCAS: moderate right common carotid artery stenosis; SE-RCCAS: severe right common carotid artery stenosis; TOS: total oxidant status; LHP: lipid hydroperoxide; 8-IP: 8-isoprostane; MDA: malondialdehyde; MCP-4: monocyte chemotactic protein-4; AA: amyloid A; hs-CRP: high-sensitivity C-reactive protein; TNF-*α*: tumor necrosis factor-*α*. *P* > 0.05 (CON group/no carotid stenosis group). ^∗^*P* < 0.001 (no carotid stenosis group/MI-RCCAS group). ^∗∗^*P* < 0.001 (MI-RCCAS group/MO-RCCAS group). ^∗∗∗^*P* < 0.001 (MO-RCCAS group/SE-RCCAS group). Group comparisons (CON group/no carotid stenosis group/MI-RCCAS group/MO-RCCAS group/SE-RCCAS group) were made using ANOVA, *P* < 0.001.

**Table 3:** Levels of biomarkers in patients with RECAS.

	CON (*n* = 61)	No carotid stenosis (*n* = 56)	MI-RECAS (*n* = 54)	MO-RECAS (*n* = 51)	SE-RECAS (*n* = 57)
TOS (*μ*mol H_2_O_2_ Eq/L)	7.3 ± 0.7	7.4 ± 0.7	8.9 ± 0.9^∗^	10.0 ± 1.2^∗∗^	12.1 ± 1.3^∗∗∗^
LHP (*μ*mol/L)	5.8 ± 0.6	5.9 ± 0.6	7.0 ± 0.7^∗^	8.5 ± 0.9^∗∗^	9.7 ± 2.3^∗∗∗^
8-IP (pg/mL)	45.2 ± 4.5	45.0 ± 4.4	59.7 ± 6.0^∗^	65.2 ± 6.4^∗∗^	82.0 ± 7.8^∗∗∗^
MDA (nmol/L)	1.7 ± 0.3	1.6 ± 0.2	3.8 ± 0.5^∗^	4.9 ± 0.6^∗∗^	7.2 ± 0.8^∗∗∗^
MCP-4 (ng/mL)	41.9 ± 4.2	42.0 ± 4.2	59.6 ± 6.0^∗^	78.5 ± 7.9^∗∗^	96.3 ± 8.9^∗∗∗^
AA (mg/L)	35.2 ± 3.5	36.1 ± 3.6	54.1 ± 5.3^∗^	75.0 ± 7.3^∗∗^	96.2 ± 8.7^∗∗∗^
hs-CRP (mg/L)	2.7 ± 0.3	2.6 ± 0.3	4.4 ± 0.5^∗^	6.9 ± 0.6^∗∗^	8.5 ± 0.7^∗∗∗^
TNF-*α* (ng/L)	20.1 ± 2.0	21.3 ± 2.1	40.4 ± 3.9^∗^	69.7 ± 7.0^∗∗^	88.6 ± 9.1^∗∗∗^

RECAS: right external carotid artery stenosis; CON: control; MI-RECAS: mild right external carotid artery stenosis; MO-RECAS: moderate right external carotid artery stenosis; SE-RECAS: severe right external carotid artery stenosis; TOS: total oxidant status; LHP: lipid hydroperoxide; 8-IP: 8-isoprostane; MDA: malondialdehyde; MCP-4: monocyte chemotactic protein-4; AA: amyloid A; hs-CRP: high-sensitivity C-reactive protein; TNF-*α*: tumor necrosis factor-*α*. *P* > 0.05 (CON group/no carotid stenosis group). ^∗^*P* < 0.001 (no carotid stenosis group/MI-RECAS group). ^∗∗^*P* < 0.001 (MI-RECAS group/MO-RECAS group). ^∗∗∗^*P* < 0.001 (MO-RECAS group/SE-RECAS group). Group comparisons (CON group/no carotid stenosis group/MI-RECAS group/MO-RECAS group/SE-RECAS group) were made using ANOVA, *P* < 0.001.

**Table 4:** Levels of biomarkers in patients with RICAS.

	CON (*n* = 61)	No carotid stenosis (*n* = 56)	MI-RICAS (*n* = 57)	MO-RICAS (*n* = 54)	SE-RICAS (*n* = 56)
TOS (*μ*mol H_2_O_2_ Eq/L)	7.3 ± 0.7	7.4 ± 0.7	13.7 ± 1.4^∗^	18.0 ± 2.0^∗∗^	26.9 ± 3.0^∗∗∗^
LHP (*μ*mol/L)	5.8 ± 0.6	5.9 ± 0.6	7.9 ± 0.7^∗^	11.5 ± 1.2^∗∗^	12.7 ± 1.4^∗∗∗^
8-IP (pg/mL)	45.2 ± 4.5	45.0 ± 4.4	69.7 ± 7.0^∗^	95.0 ± 9.2^∗∗^	106.4 ± 10.3^∗∗∗^
MDA (nmol/L)	1.7 ± 0.3	1.6 ± 0.2	3.8 ± 0.4^∗^	6.5 ± 1.3^∗∗^	9.2 ± 1.2^∗∗∗^
MCP-4 (ng/mL)	41.9 ± 4.2	42.0 ± 4.2	69.7 ± 7.0^∗^	88.5 ± 8.2^∗∗^	116.3 ± 11.6^∗∗∗^
AA (mg/L)	35.2 ± 3.5	36.1 ± 3.6	54.1 ± 5.9^∗^	85.0 ± 9.0^∗∗^	109.2 ± 10.3^∗∗∗^
hs-CRP (mg/L)	2.7 ± 0.3	2.6 ± 0.3	5.2 ± 0.5^∗^	7.9 ± 0.8^∗∗^	11.0 ± 1.1^∗∗∗^
TNF-*α* (ng/L)	20.1 ± 2.0	21.3 ± 2.1	50.4 ± 5.3^∗^	79.7 ± 8.0^∗∗^	98.6 ± 9.2^∗∗∗^

RICAS: right internal carotid artery stenosis; CON: control; MI-RICAS: mild right internal carotid artery stenosis; MO-RICAS: moderate right internal carotid artery stenosis; SE-RICAS: severe right internal carotid artery stenosis; TOS: total oxidant status; LHP: lipid hydroperoxide; 8-IP: 8-isoprostane; MDA: malondialdehyde; MCP-4: monocyte chemotactic protein-4; AA: amyloid A; hs-CRP: high-sensitivity C-reactive protein; TNF-*α*: tumor necrosis factor-*α*. *P* > 0.05 (CON group/no carotid stenosis group). ^∗^*P* < 0.001 (no carotid stenosis group/MI-RICAS group). ^∗∗^*P* < 0.001 (MI-RICAS group/MO-RICAS group). ^∗∗∗^*P* < 0.001 (MO-RICAS group/SE-RICAS group). Group comparisons (CON group/no carotid stenosis group/MI-RICAS group/MO-RICAS group/SE-RICAS group) were made using ANOVA, *P* < 0.001.

**Table 5:** Levels of biomarkers in patients with carotid artery severe stenosis.

	CON (*n* = 61)	No carotid stenosis (*n* = 56)	SE-RCCAS+SE-RECAS (*n* = 58)	SE-RCCAS+SE-RICAS (*n* = 55)	SE-RCCAS+SE-RECAS+SE-RICAS (*n* = 52)
TOS (*μ*mol H_2_O_2_ Eq/L)	7.3 ± 0.7	7.4 ± 0.7	17.3 ± 1.6^∗^	33.9 ± 2.4^∗∗^	46.9 ± 3.6^∗∗∗^
LHP (*μ*mol/L)	5.8 ± 0.6	5.9 ± 0.6	11.7 ± 1.2^∗^	18.3 ± 1.6^∗∗^	24.7 ± 2.3^∗∗∗^
8-IP (pg/mL)	45.2 ± 4.5	45.0 ± 4.4	73.7 ± 7.3^∗^	115.0 ± 10.5^∗∗^	146.4 ± 13.6^∗∗∗^
MDA (nmol/L)	1.7 ± 0.3	1.6 ± 0.2	4.0 ± 0.4^∗^	7.5 ± 0.6^∗∗^	10.2 ± 0.9^∗∗∗^
MCP-4 (ng/mL)	41.9 ± 4.2	42.0 ± 4.2	79.7 ± 6.8^∗^	138.5 ± 12.8^∗∗^	176.3 ± 16.4^∗∗∗^
AA (mg/L)	35.2 ± 3.5	36.1 ± 3.6	60.9 ± 5.9^∗^	86.0 ± 7.6^∗∗^	110.2 ± 10.1^∗∗∗^
hs-CRP (mg/L)	2.7 ± 0.3	2.6 ± 0.3	5.4 ± 0.4^∗^	8.4 ± 0.6^∗∗^	12.0 ± 0.9^∗∗∗^
TNF-*α* (ng/L)	20.1 ± 2.0	21.3 ± 2.1	49.4 ± 3.9^∗^	79.7 ± 6.5^∗∗^	118.6 ± 10.8^∗∗∗^

CON: control; SE-RCCAS: severe right common carotid artery stenosis; SE-RECAS: severe right external carotid artery stenosis; SE-RICAS: severe right internal carotid artery stenosis; TOS: total oxidant status; LHP: lipid hydroperoxide; 8-IP: 8-isoprostane; MDA: malondialdehyde; MCP-4: monocyte chemotactic protein-4; AA: amyloid A; hs-CRP: high-sensitivity C-reactive protein; TNF-*α*: tumor necrosis factor-*α*. *P* > 0.05 (CON group/no carotid stenosis group). ^∗^*P* < 0.001 (no carotid stenosis group/SE-RCCAS+SE-RECAS group). ^∗∗^*P* < 0.001 (SE-RCCAS+SE-RECAS group/SE-RCCAS+SE-RICAS group). ^∗∗∗^*P* < 0.001 (SE-RCCAS+SE-RICAS group/SE-RCCAS+SE-RECAS+SE-RICAS group). Group comparisons (CON group/no carotid stenosis group/SE-RCCAS+SE-RECAS group/SE-RCCAS+SE-RICAS group/SE-RCCAS+SE-RECAS+SE-RICAS group) were made using ANOVA, *P* < 0.001.

**Table 6:** Levels of biomarkers in patients with carotid artery severe stenosis and severe coronary artery stenosis.

	CON (*n* = 61	No carotid stenosis (*n* = 56)	SE-RCCAS+SOVCS (*n* = 51)	SE-RECAS+STVCS (*n* = 50)	SE-RICAS+SMVCS (*n* = 54)
TOS (*μ*mol H_2_O_2_ Eq/L)	7.3 ± 0.7	7.4 ± 0.7	18.6 ± 1.5^∗^	39.0 ± 3.6^∗∗^	48.9 ± 4.5^∗∗∗^
LHP (*μ*mol/L)	5.8 ± 0.6	5.9 ± 0.6	12.0 ± 0.9^∗^	19.5 ± 2.0^∗∗^	28.6 ± 3.1^∗∗∗^
8-IP (pg/mL)	45.2 ± 4.5	45.0 ± 4.4	83.9 ± 7.2^∗^	135.0 ± 12.5^∗∗^	159.4 ± 14.8^∗∗∗^
MDA (nmol/L)	1.7 ± 0.3	1.6 ± 0.2	5.8 ± 0.4^∗^	9.0 ± 0.7^∗∗^	11.3 ± 1.2^∗∗∗^
MCP-4 (ng/mL)	41.9 ± 4.2	42.0 ± 4.2	87.9 ± 7.9^∗^	149.5 ± 13.2^∗∗^	190.3 ± 18.6^∗∗∗^
AA (mg/L)	35.2 ± 3.5	36.1 ± 3.6	64.1 ± 5.4^∗^	86.0 ± 7.4^∗∗^	120.2 ± 11.3^∗∗∗^
hs-CRP (mg/L)	2.7 ± 0.3	2.6 ± 0.3	5.9 ± 0.5^∗^	10.4 ± 0.9^∗∗^	14.5 ± 1.3^∗∗∗^
TNF-*α* (ng/L)	20.1 ± 2.0	21.3 ± 2.1	53.4 ± 5.2^∗^	99.7 ± 10.1^∗∗^	140.6 ± 13.0^∗∗∗^

CON: control; SE-RCCAS: severe right common carotid artery stenosis; SOVCS: severe one-vessel coronary stenosis; SE-RECAS: severe right external carotid artery stenosis; STVCS: severe two-vessel coronary stenosis; SE-RICAS: severe right internal carotid artery stenosis; SMVCS: severe multivessel coronary stenosis; TOS: total oxidant status; LHP: lipid hydroperoxide; 8-IP: 8-isoprostane; MDA: malondialdehyde; MCP-4: monocyte chemotactic protein-4; AA: amyloid A; hs-CRP: high-sensitivity C-reactive protein; TNF-*α*: tumor necrosis factor-*α*. *P* > 0.05 (CON group/no carotid stenosis group). ^∗^*P* < 0.001 (no carotid stenosis group/SE-RCCAS+OVCS group). ^∗∗^*P* < 0.001 (SE-RCCAS+OVCS group/SE-RECAS+TVCS group). ^∗∗∗^*P* < 0.001 (SE-RECAS+TVCS group/SE-RICAS+MVCS group). Group comparisons (CON group/no carotid stenosis group/SE-RCCAS+OVCS group/SE-RECAS+TVCS group/SE-RICAS+MVCS group) were made using ANOVA, *P* < 0.001.

**Table 7:** The incidences of severe right carotid stenosis and coronary artery stenosis.

Age groups (years)	MI-RCS (*n* = 170)	MO-RCS (*n* = 160)	SE-RAS (*n* = 166)	SE-RAS+SMVCS (*n* = 106)
65-70, *n* (%)	80 (47.0)	70 (41.7)	40 (24.0)	22 (20.7)
75-80, *n* (%)	60 (34.2)^∗^	50 (33.2)^∗^	50 (33.1)^∗^	31 (32.2)^∗^
81-86, *n* (%)	30 (18.6)^∗∗^	40 (25.0)^∗∗^	76 (42.7)^∗∗^	53 (47.0)^∗∗^

MI-RCS: mild right carotid stenosis; MO-RCS: moderate right carotid stenosis; SE-RAS: severe right arotid stenosis; SE-RAS+SMVCS: severe right arotid stenosis+severe multivessel coronary stenosis. ^∗^*P* < 0.05 (65-70 age group/75-80 age group). ^∗∗^*P* < 0.05 (75-80 age group/81-86 age group). Group comparisons (65-70 age group/75-80 age group/81-86 age group) were made using ANOVA, *P* <0.05.

**Table 8:** Multiple regression analysis to evaluate risk predictors for carotid and coronary artery stenosis.

TOS	5.30	1.45-18.65	0.002
LHP	3.19	1.32-2.97	0.01
8-IP	2.50	1.30-3.14	0.04
MDA	4.78	1.46-19.86	0.001
MCP-4	2.36	1.35-4.20	0.03
AA	3.07	1.31-2.99	0.04
Hs-CRP	4.92	1.62-12.05	0.001
TNF-*α*	5.81	1.49-19.83	0.001

TOS: total oxidant status; LHP: lipid hydroperoxide; 8-IP: 8-isoprostane; MDA: malondialdehyde; MCP-4: monocyte chemotactic protein-4; AA: amyloid A; hs-CRP: high-sensitivity C-reactive protein; TNF-*α*: tumor necrosis factor-*α*.

## Data Availability

All relevant data are within this research paper. All data used to support the findings of the research are available from the corresponding author on reasonable. No additional data are available.
